# The Emerging Roles of Vacuolar-Type ATPase-Dependent Lysosomal Acidification in Cardiovascular Disease

**DOI:** 10.3390/biom15040525

**Published:** 2025-04-03

**Authors:** Yan-Yan Chen, Cai-Xia Liu, Hai-Xin Liu, Shi-Yuan Wen

**Affiliations:** 1 School of Medicine, Jiangsu University, Zhenjiang 212013, China; 2 College of Traditional Chinese Medicine and Food Engineering, Shanxi University of Chinese Medicine, Taiyuan 030024, China; liucx1104@sxtcm.edu.cn (C.-X.L.); liuhaixin@sxtcm.edu.cn (H.-X.L.); 3 College of Basic Medical Sciences, Shanxi Medical University, Taiyuan 030001, China

**Keywords:** vacuolar-type ATPase, lysosomal acidification, cardiovascular disease

## Abstract

The vacuolar-type ATPase (V-ATPase) is a multi-subunit enzyme complex that maintains lysosomal acidification, a critical process for cellular homeostasis. By controlling the pH within lysosomes, V-ATPase contributes to overall cellular homeostasis, helping to maintain a balance between the degradation and synthesis of cellular components. Dysfunction of V-ATPase impairs lysosomal acidification, leading to the accumulation of undigested materials and contributing to various diseases, including cardiovascular diseases (CVDs) like atherosclerosis and myocardial disease. Furthermore, V-ATPase’s role in lysosomal function suggests potential therapeutic strategies targeting this enzyme complex to mitigate cardiovascular disease progression. Understanding the mechanisms by which V-ATPase influences cardiovascular pathology is essential for developing novel treatments aimed at improving outcomes in patients with heart and vascular diseases.

## 1. Introduction

Lysosomal acidification refers to the process by which the interiors of lysosomes are maintained at a lower pH than the cytoplasm, typically around 4.5 to 5.0 [1,2]. This acidic environment is crucial for several cellular processes, including enzyme activation, degradation of biomolecules, autophagy, endocytosis and phagocytosis, pathogen defense, biosynthesis, and sorting [3]. Recent research suggests that lysosomal acidification may also influence mitochondrial function, possibly by affecting the transport of certain molecules between the two organelles [4]. Impaired lysosomal acidification can lead to various diseases, including cardiovascular disease (CVD). In summary, lysosomal acidification is a critical process that enables the cell to efficiently recycle its components, degrade engulfed materials, and maintain cellular health. The maintenance of this acidic environment is essential for the proper functioning of lysosomes and the overall homeostasis of the cell.

Vacuolar-type ATPase (V-ATPase) is a critical component in the process of lysosomal acidification due to its role as a proton pump [5]. V-ATPase actively transports protons (H^+^ ions) from the cytoplasm into the lysosome, which is the primary mechanism for lowering the pH within the lysosome. The energy required for V-ATPase comes from the hydrolysis of ATP (adenosine triphosphate), which is the primary energy currency of the cell. By maintaining an acidic environment within lysosomes, V-ATPase ensures that the lysosomal enzymes (acid hydrolases) can function optimally. These enzymes are most active at a low pH and are essential for breaking down various cellular waste materials. The activity of V-ATPase can be regulated, which in turn regulates the pH within lysosomes. This regulation is important for controlling the rate of lysosomal degradation and for preventing damage to the cell from uncontrolled enzyme activity. By controlling the pH within lysosomes, V-ATPase contributes to overall cellular homeostasis, helping to maintain a balance between the degradation and synthesis of cellular components [6]. Mutations or defects in V-ATPase can lead to a group of disorders known as lysosomal storage diseases [7]. In these conditions, the inability to maintain an acidic lysosomal pH impairs the degradation of cellular waste, leading to the accumulation of undigested materials and causing various symptoms. In addition, V-ATPase is also involved in the transport of certain molecules across the lysosomal membrane, which is facilitated by the proton gradient created by the pump [8].

V-ATPase is the key enzyme that enables lysosomal acidification, which is essential for the proper functioning of lysosomes and for maintaining cellular health and homeostasis. In the context of CVDs, the role of V-ATPase-dependent lysosomal acidification is particularly intriguing. Understanding the intricate details of V-ATPase function and regulation could, therefore, offer valuable insights into the pathogenesis of CVDs and potentially lead to the development of novel therapeutic strategies. This profound discourse aims to delve into the multifaceted roles of lysosomes, the intricate regulatory mechanisms of V-ATPase, and their implications in the realm of cardiovascular health and disease.

## 2. Lysosome Function

### 2.1. Lysosomes and Their Role in Cellular Degradation

Lysosomes exert the primary function in cellular degradation and the recycling of materials because lysosomes contain a variety of hydrolytic enzymes that are capable of breaking down various biomolecules, including proteins, lipids, nucleic acids, and carbohydrates [9]. The enzymes within lysosomes are active at an acidic pH, which is maintained by V-ATPase in the lysosomal membrane that pumps H^+^ into the lysosome [10]. This acidic environment is crucial for the optimal activity of the enzymes.

One of the primary functions of lysosomes is autophagy, a process that encloses damaged organelles or proteins in a double-membrane structure called an autophagosome, which then fuses with a lysosome [9]. Lysosomes also play a role in endocytosis, where the cell takes in extracellular material. After the material is internalized in vesicles, these vesicles can fuse with lysosomes to degrade the engulfed substances [11]. In immune cells, such as macrophages, lysosomes are involved in the destruction of pathogens. The cell engulfs the pathogen in a process called phagocytosis, and the pathogen-containing vesicle fuses with a lysosome, where it is degraded. Lysosomes are also involved in programmed cell death, or apoptosis. During this process, lysosomal enzymes can be released into the cytoplasm, leading to the breakdown of cellular components and the eventual death of the cell. The activity of lysosomes is tightly regulated to prevent damage to the cell [12]. In lysosomal storage diseases, specific enzymes are missing or dysfunctional, leading to the accumulation of undigested material within the lysosomes, which can cause cellular dysfunction and various symptoms [13]. Lysosomes are essential for maintaining cellular health by recycling cellular components, degrading foreign substances, and participating in the cell’s response to stress and damage.

### 2.2. The Importance of Maintaining Acidic pH Within Lysosomes for Proper Enzymatic Activity

The acidic pH within lysosomes is crucial for their proper function and enzymatic activity. In humans, the pH of lysosomes is generally around 4.5 to 5.0, which is significantly lower than the neutral pH found in the cytoplasm (around 7.3 to 7.4) [1,2]. The acidic environment within lysosomes helps to maintain the stability of the enzymes [14]. The acidic pH within lysosomes can also play a role in regulating enzyme activity and catalyzing the breakdown of various biomolecules effectively. Some enzymes may require protonation at low pH to become active, while others may be inhibited by higher pH levels. The acidic pH can also influence the specificity of the enzymes for their substrates. Certain substrates may be more accessible or reactive at lower pH levels, allowing for more efficient degradation [15]. The lysosomal membrane itself is composed of lipids and proteins that are adapted to function in an acidic environment. The maintenance of an acidic pH helps to preserve the integrity of the lysosomal membrane, preventing the leakage of enzymes into the cytoplasm, which could be harmful to the cell [16]. The acidic pH within lysosomes can also have antimicrobial properties. When pathogens are engulfed by the cell through phagocytosis, the acidic environment of the lysosome can help to inactivate or kill the pathogen, in addition to the action of the lysosomal enzymes [9,17]. The V-ATPase in the lysosomal membrane that maintains the acidic pH also creates a proton gradient across the membrane. This gradient can be used to drive the transport of certain molecules into or out of the lysosome, facilitating the selective uptake or release of substances [18,19].

**Figure 1:**
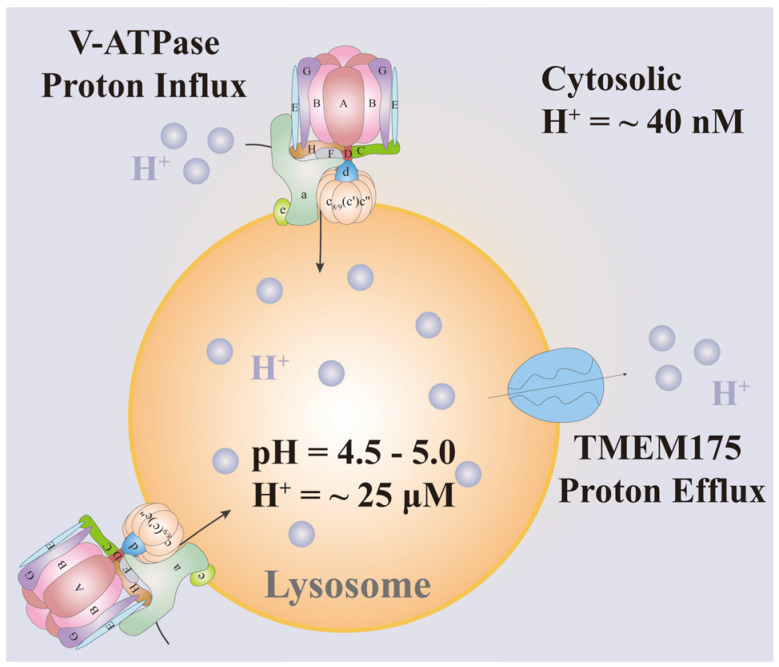
V-ATPase and TMEM175 maintain the acidic balance of the lysosome. V-ATPase regulates H^+^ uptake into lysosomes, whereas TMEM175 regulates H^+^ release from lysosomes. Thus, the intracellular compartment pH of the lysosome is maintained from 4.5 to 5.0.

The acidic pH within lysosomes is essential for the proper functioning of the enzymes contained within, the stability and integrity of the lysosomal membrane, and the overall health of the cell. Any disruption to this pH balance can lead to impaired lysosomal function and contribute to various pathological conditions [20]. In eukaryotic cells, the transporter responsible for controlling lysosomal acidification is V-ATPase [5], and TMEM175 regulates lysosomal H^+^ release [21], so that V-ATPase and TMEM175 maintain the acidic balance of the lysosome (Figure 1). A recent study has shown that human lysosomal-associated membrane proteins (LAMPs) can be complexed with TMEM175, which can counterbalance the proton influx of V-ATPase [22].

## 3. V-ATPase and Its Structure

**Figure 2:**
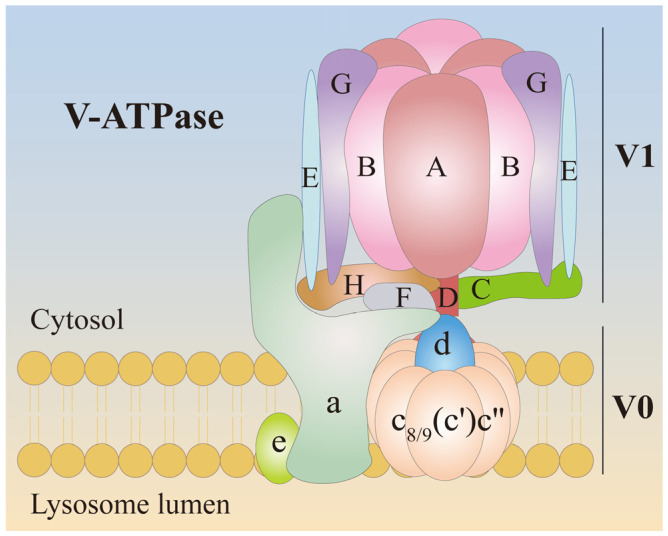
The structure of V-ATPase. V-ATPase consists of two main parts: a V0 sector that spans the membrane and a V1 sector that protrudes into the cytoplasm. V1 is composed of subunits A through H in a stoichiometry of A_3_B_3_CDE_3_FG_3_H, while V0 is composed of subunits a, c, c″, d, e, in a stoichiometry of ac_9_c″de, as well as ATP6AP1 and ATP6AP2.

V-ATPase consists of two main parts: a V0 sector that spans the membrane and a V1 sector that protrudes into the cytoplasm. The V-ATPase complex includes various subunits, some of which have isoforms that can be tissue-specific, allowing for organ-specific regulation [23]. V1 is composed of subunits A through H in a stoichiometry of A_3_B_3_CDE_3_FG_3_H, while V0 is composed of subunits a, c, c″, d, e, in a stoichiometry of ac_9_c″de, as well as ATP6AP1 and ATP6AP2, each with specific roles in the complex’s function (Figure 2). The V0 sector contains a c-ring structure that allows protons to pass through, while the V1 sector contains ATP hydrolysis sites that provide the energy for proton transport [24].

By pumping protons into the lysosome, the acidic environment for the optimal activity of lysosomal enzymes is accomplished based on V-ATPase concentration, selection of V-ATPase isoforms, and association and dissociation of the V0 and V1 sectors [2,10]. The activity of V-ATPase is tightly regulated to ensure that the organelles maintain their specific pH levels. This regulation involves various mechanisms, including the reversible dissociation of the V1 and V0 sectors, which can modulate the pump’s activity [25]. Without the acidification provided by V-ATPase, these enzymes would not function properly, and lysosomal degradation would be impaired [1]. Mutations, mislocalizations, or dysregulations of V-ATPase are associated with various diseases, including CVD [26], neurodegenerative disease [6], cancer [27], and so on.

In summary, V-ATPase is a key regulator of lysosomal acidification, and its function is essential for the proper degradation of cellular and extracellular materials within lysosomes. Understanding the regulation and function of V-ATPase on the lysosome is important for insights into cellular physiology and the development of treatments for CVD.

## 4. Regulation of V-ATPase

The regulation of V-ATPase activity is a complex and multifaceted process that ensures the enzyme functions correctly in response to cellular needs, such as autophagic processes [28]. The activity of V-ATPase can be regulated by various mechanisms, including regulated assembly of V-ATPase, regulated trafficking, adjustments in coupling efficiency, post-translational modification, regulation of subunit expression levels, and changes in counterion conductance. This regulation allows the cell to modulate the lysosomal pH in response to different physiological conditions. Regulation of V-ATPase is summarized in Table 1.

### 4.1. Regulated Assembly

**Figure 3:**
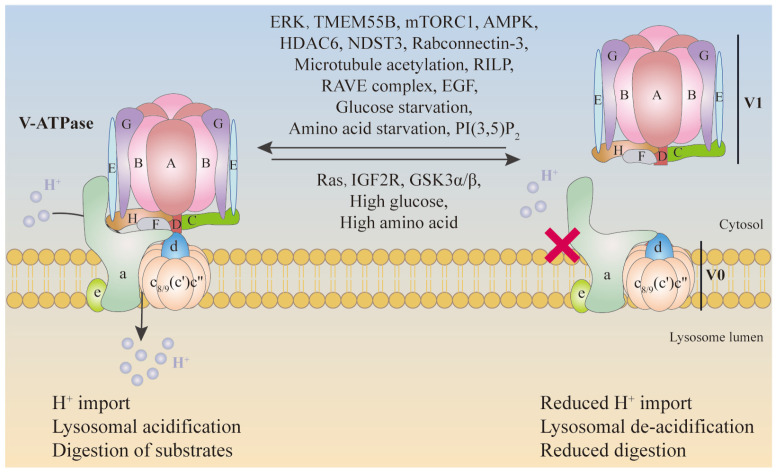
The assembly regulation of V-ATPase in mammalian cells. ERK, TMEM55B, mTORC1, AMPK, HDAC6, NDST3, Rabconnectin-3, microtubule acetylation, RILP, RAVE complex, EGF, glucose starvation, amino acid starvation, and PI(3,5)P2 can promote the assembly of V1 and V0 sectors to reduce H^+^ import. On the contrary, Ras, IGF2R, GSK3α/β, high glucose, and high amino acids can inhibit the assembly of V1 and V0 sectors, maintaining H^+^ import.

Among the most important forms of V-ATPase regulation is the reversible dissociation of the V-ATPase complex into its component V1 and V0 sectors, a process termed regulated assembly. The V-ATPase complex is composed of multiple subunits that must be assembled correctly to function. The assembly process can be regulated by cellular signals, ensuring that V-ATPase is only fully assembled and active when needed, as shown in Figure 3.

In yeast, disruption of the binding between aldolase and the B subunit of V-ATPase results in the disassembly and malfunction of V-ATPase [30]. However, in the endosome/lysosome of HeLa cells, aldolase engages with three specific subunits of the V-ATPase complex: the transmembrane a subunit within the V0 sector and the soluble E and B subunits of the V1 sector [29]. Importantly, aldolase knockdown causes subcellular redistribution of V-ATPase-driven acidic vesicles but does not change the acidification capacity of endosomal/lysosomal compartments [29]. This interaction is modulated by glucose, hinting at a potential role for aldolase as a glucose sensor that could influence the assembly and disassembly of V-ATPase, thus modulating its activity. Importantly, it has been discovered that the regulation of V-ATPase’s assembly and disassembly processes is independent of aldolase’s enzymatic function, underscoring the importance of the physical interaction within the V-ATPase/aldolase complex [30]. Furthermore, the dynamics of V-ATPase assembly and disassembly are also under the control of the Ras/cAMP/PKA signaling pathway [31]. Notably, Ras overexpression results in a decrease in aldolase binding to V-ATPase [31], highlighting the intricate regulatory mechanisms at play.

The Rab-interacting lysosomal protein (RILP) skillfully orchestrates the recruitment of the ATP6V1G1 subunit of the V-ATPase to the membranes of late endosomes and lysosomes and simultaneously ensures the stability of ATP6V1G1 [39]. This dual role allows RILP to intricately modulate the V-ATPase assembly, which is vital for preserving the luminal pH and enzymatic activity of these organelles [64]. In yeast, the regulator of the H^+^-ATPase of vacuoles and endosomes (RAVE) complex facilitates V-ATPase assembly and regulates its function on organelle membranes. In higher eukaryotes, Rabconnectin-3 complexes play a similar role in assembling and regulating the V-ATPase complex to control the acidification of organelles such as lysosomes [37,65,66]. The dissociation process of the V-ATPase complex is independent of de novo protein synthesis and depends on the integrity of microtubules [67]. Loss of NDST3 enhances the assembly of the V-ATPase holoenzyme on the lysosomal membrane via microtubule acetylation, resulting in increased lysosome acidification [32]. Moreover, HDAC6 mediates V-ATPase assembly to promote lysosomal acidification [43].

Under various stimuli, the V-ATPase complex is capable of undergoing reversible dissociation into its V1 and V0 sectors, effectively halting ATP-driven proton translocation. Within the framework of yeast metabolism, such reversible dissociation is triggered by swift glucose exhaustion. Remarkably, a mere 5 min period of glucose deprivation can lead to the disassembly of approximately 70% of the V-ATPase complexes into their constituent V1 and V0 subcomplexes [33]. Conversely, the reintroduction of glucose promotes a swift and efficient reassembly of the enzyme from these pre-existing subcomplexes, thus restoring its functional integrity [34]. However, research on mammalian cells has shown that acute glucose starvation can enhance the assembly of V-ATPase via the AMPK and PI3K/Akt signaling pathways, contrary to causing its disassembly [35]. The distinct response patterns of mammalian cells and yeast to glucose starvation can be attributed to the following factors. Firstly, energy source variation: mammalian cells and yeast differ in energy sources and metabolic pathways. Mammalian cells rely more on a constant glucose supply, while yeast can utilize various carbon sources with greater metabolic flexibility. Secondly, cell type and function variation: mammalian cells, part of multicellular organisms, need to maintain complex physiological functions. Yeast, being single-celled, focuses on rapid environmental adaptation. Thirdly, evolutionary strategy variation: mammalian cells and yeast have evolved different mechanisms to cope with glucose deficiency. Mammalian cells tend to inhibit non-essential energy consumption and protect crucial functions to survive. Yeast adjusts its metabolism quickly and may enter a resting phase to adapt. Therefore, glucose has opposite effects on V-ATPase assembly/disassembly in mammalian cells and yeast.

Starvation for amino acids has been demonstrated to enhance the assembly of the V-ATPase complex, and the subsequent reintroduction of amino acids can counteract this assembly [36]. TMEM55B interacts with V-ATPase, resulting in the assembly of the V-ATPase complex in the lysosomal membrane lipid rafts and the subsequent activation of mTORC1 [41]. The epidermal growth factor (EGF) stimulates the recruitment of the extrinsic V1 subunits of the V-ATPase to the endosomes in rat liver cells [38]. Low-dose insulin-like growth factor 2 (IGF2) activates the IGF2 receptor (IGF2R) that induces Dnmt3a-mediated DNA methylation by activating GSK3α/β and subsequently blocks expression and assembly of V-ATPase [42].

These discoveries underscore the significance of the V1/V0 complex’s reversible dissociation as a potent regulatory mechanism for modulating V-ATPase activity in response to cellular signals. The precise regulation of V-ATPase assembly is vital for its cellular activity, ensuring the enzyme fulfills its role in acidifying intracellular vesicles, organelles, and the extracellular milieu—a process critical for preserving eukaryotic cellular homeostasis. The V-ATPase’s capacity to modulate lysosomal pH is particularly significant, as it opens potential avenues for developing novel therapeutic strategies against CVDs. Gaining a deeper understanding of how V-ATPase assembly and regulation are orchestrated can inform the creation of targeted interventions that leverage this proton pump’s essential function for medical advancement.

### 4.2. Regulated Trafficking

The localization of V-ATPase within the cell can also be a regulatory mechanism. V-ATPase is found in the membranes of various organelles, including endosomes, lysosomes, and the trans-Golgi network, where it functions to acidify the lumen of these compartments. The localization of V-ATPase within the cell is not random; it is a highly regulated process that ensures the enzyme’s optimal function in the acidic environments necessary for various cellular processes such as protein sorting, membrane trafficking, and the degradation of macromolecules. Proper localization is essential for V-ATPase’s role in these processes, as its activity is tightly linked to its position within the cell. This sets the stage for exploring the intricate details of V-ATPase localization and its implications for cellular function and human disease.

V-ATPase can be transported to different cellular membranes, which are controlled by isoforms of subunit a. The subunit a of the V-ATPase in yeast is unique as it has multiple isoforms, which are encoded by the VPH1 and STV1 genes [68,69]. Vph1p and Stv1p each possess N-terminal targeting sequences; V-ATPases that include Vph1p are directed to vacuoles, whereas those incorporating Stv1p are steered toward the Golgi apparatus [70]. In mammals, there are four isoforms of the subunit a, encoded by the ATP6V0a1, ATP6V0a2, TCIRG1, and ATP6V0a4 genes [71]. The a1 and a2 isoforms are predominantly associated with intracellular membranes, although a1 also plays a role in the presynaptic plasma membrane of nerve terminals [72,73,74]. The a3 isoform is located in intracellular organelles such as lysosomes, phagosomes, and secretory granules, and it targets the plasma membrane of osteoclasts [75,76,77,78]. Undifferentiated osteoclast precursor cells are found in late endosomes and lysosomes, and the a3-containing V-ATPases in these cells are translocated to the plasma membrane during their differentiation into mature osteoclasts [75]. The a1-a3 isoforms have a broad tissue distribution, whereas the a4 isoform appears to be restricted to the kidney, epididymis, and inner ear [79,80,81]. V-ATPases that contain the a4 isoform are positioned in the apical plasma membrane of renal intercalated cells and epididymal clear cells, where they are involved in urine acidification and sperm maturation, respectively [79,81]. N-glycosylation plays a crucial role in the stability of ATP6V0a4, as well as in the assembly and translocation of V-ATPase to the plasma membrane [82]. PSEN1 plays a crucial role in facilitating the N-glycosylation process of the V0a1 subunit within the endoplasmic reticulum. In the absence of PSEN1, the glycosylation of V0a1 is significantly disrupted, which hinders the efficient transport of the under-glycosylated V0a1 to the lysosomes. Consequently, this leads to a detrimental effect on the functionality of the V-ATPase enzyme and the essential process of lysosomal acidification [40].

In response to the depletion of amino acids, PAT2 relocates from the plasma membrane to the lysosomes, facilitating the assembly of the lysosomal V-ATPase complex by bringing cytosolic V1 subunits to the lysosomal membrane, which in turn compromises lysosomal acidification [49]. Beyond the targeting of V-ATPase to specific membranes through isoform composition, certain polarized cells have the capacity to swiftly modulate the plasma membrane density of V-ATPase. Cytoplasmic membranes and intracellular vesicles with a high density of V-ATPase may fuse, leading to adjustments in the proton pump’s abundance [83]. Certain specialized cells, such as the interdental cells of the ear, the epithelial cells of the nose, and the visual or renal proximal tubules, also express V-ATPase on their plasma membranes, participating in the regulation of extracellular pH [5,84,85,86]. The localization of V-ATPase to the plasma membrane is controlled by the reversible exocytosis and endocytosis of vesicles containing a high density of V-ATPase [87]. In renal intercalated cells and epididymal clear cells, exposure to alkaline pH triggers an increase in bicarbonate uptake, which in turn activates PKA via the bicarbonate-sensitive adenylyl cyclase [88]. The elevation of cAMP promotes the insertion of V-ATPase into the apical membrane, a process in which PKA-dependent phosphorylation of the subunit a plays a pivotal role [46,47,48]. Soluble adenylyl cyclase (sAC), a broadly expressed generator of cAMP, serves as a cellular sensor for physiological pH levels [89]. Within epithelial cells that emit protons, sAC orchestrates the pH-dependent trafficking of V-ATPase to the luminal surface, a process crucial for the enzyme’s function. Evidence from both genetic and pharmacological studies indicates that sAC is indispensable for the acidification of lysosomes. In the absence of sAC, V-ATPase fails to localize correctly to the lysosomes, resulting in incomplete acidification, diminished lysosomal degradative capabilities, and the accumulation of autolysosomes [90]. On the other hand, AMPK counteracts the PKA-induced aggregation of V-ATPase at the apical membrane in renal and epididymal cells [44]. Within the renal cells, this phenomenon is contingent upon the direct phosphorylation of subunit A by AMPK [45]. Importantly, these investigations utilize the V1 subunit’s localization as an indicator of the intact enzyme’s transport, suggesting that both AMPK and PKA may concurrently influence the assembly of V-ATPase in these cell types.

In conclusion, the localization of V-ATPase within the cell is a meticulously orchestrated process that is integral to its diverse functions in cellular physiology. The strategic placement of V-ATPase ensures not only the optimal function of the enzyme itself but also influences broader cellular processes, including membrane trafficking, protein processing, and the regulation of cellular signaling pathways. The enzyme’s localization may serve as a novel target for modulating its activity in cardiovascular disorders where V-ATPase function is dysregulated. Therefore, the study of V-ATPase localization is not only a fascinating exploration into cellular logistics but also a gateway to developing new strategies for treating CVDs.

### 4.3. Other Forms of Regulation

Other regulatory mechanisms for V-ATPases have been identified, which include adjustments in coupling efficiency, protein–protein interaction, post-translational modification, regulation of subunit expression levels, and changes in counterion conductance.

One such mechanism to modulate V-ATPase activity within living organisms involves tweaking the balance between proton transport and ATP hydrolysis, commonly referred to as coupling efficiency. It has been observed that specific mutations in distinct non-homologous areas of subunit A can enhance this coupling efficiency, hinting at the possibility that native yeast V-ATPases might not be operating at their fullest potential in terms of coupling [59]. Moreover, the coupling efficiency is also subject to the influence of subunit a isoforms; V-ATPases that incorporate vph1p exhibit a coupling efficiency that is substantially higher—by a margin of 4-5 times—compared to those that contain Stv1p [60]. Intriguingly, recent research has pointed out that the cytosolic half-channel entryway in subunit A presents a negatively charged surface in Vph1p versus a positively charged surface in Stv1p [91]. Further investigation is necessary to ascertain if this property plays a role in the variance in coupling efficiency observed between V-ATPases with Vph1p and those with Stv1p.

ZNRF2 interacts with V-ATPase and positively regulates its function, thereby maintaining lysosomal acidity [62]. Lysosomal acidification is essential for autophagy, a process that helps maintain cellular homeostasis by recycling damaged components, so V-ATPase dysfunction can lead to impaired autophagy. ITM2A negatively modulates autophagy flux through its interaction with V-ATPase, which is attributed to the suppression of lysosomal function due to ITM2A’s interference with the enzymatic activity of v-ATPase [61]. In addition, chloroquine induces endolysosomal LC3 lipidation through a mechanism that is dependent on V-ATPase activity, and similarly, the osmotic stress-induced activation of endolysosomal LC3 lipidation is also contingent upon the functional integrity of V-ATPase [92].

V-ATPase subunits can undergo post-translational modifications, which can alter the activity of the enzyme. The formation or cleavage of an intramolecular disulfide bond between cysteine residues near the catalytic site of an enzyme [93], such as the disulfide bond formed between cysteine 254 and cysteine 532 in the A subunit of bovine V-ATPase, can suppress the activation of the enzyme [58]. This inhibitory effect can be reversed by disulfide exchange with a third conserved cysteine residue [93].

The abundance of V-ATPase is regulated by some of its subunits [75]. For instance, FBXO9 promotes the ubiquitination of subunit ATP6V1A of V-ATPase, which hinders the V-ATPase assembly [52]. Importantly, the V-ATPase subunit can interact with various proteins that can modulate V-ATPase activity. For example, Lamtor5 is physically associated with ATP6V1A in promoting the V0/V1 assembly, facilitating lysosome acidification [53]. In addition, Milosevic et al. have proposed a regulatory model for synaptic vesicle V-ATPase [94]. It is currently believed that the activity of V-ATPase in clathrin-coated vesicles is inhibited by the clathrin coat. Upon removal of the coat from CCVs, the V-ATPase becomes functional, acidifying the uncoated vesicles [51]. This regulation of V-ATPase activity may be an important mechanism for the appropriate timing of synaptic vesicle refilling. Moreover, ATP6V1C1/ATP6V1B2 upregulates V-ATPase function to drive increased lysosomal acidification [95]. Prosapogenin A significantly upregulates three key functional subunits of V-ATPase, ATP6V1A, ATP6V1B2, and ATP6V0C, resulting in lysosomal over-acidification that exacerbates lysosomal damage [57]. DDRGK1 inhibits ubiquitin–proteasome-mediated degradation of V-ATPase subunits (including ATP6V0d1 and ATP6V1A) and maintains the stable expression of them, which leads to a normal function of lysosomes, promotes the fusion of autophagosomes and lysosomes, and the subsequent autophagic degradation [54]. A report indicates that miR-1 coordinately regulates lysosomal V-ATPase and its biogenesis by directly downregulating the subunit vha-13/ATP6V1A via its 3′ untranslated region (3′UTR) [55]. RORα induces the transcription of genes related to lysosomal function, such as the V-ATPase subunit ATP6V1G1; targeting RORα may be a potential therapeutic strategy to restore lysosomal acidification [56]. TFEB orchestrates the fusion of autophagosomes with lysosomes primarily through the bridging of STX17 and VAMP8, which is largely dependent on the scaffold protein ATP6V0c [96]. This process is essential for the induction of autophagy flux integrity. The voltage-gated calcium channel Cav2.3 has been shown to directly interact with the G1 subunit of the V-ATPase [97]. CAPS1 may regulate the assembly of the V-ATPase by conveying calcium signals through Rabconnectin-3 [98]. Big1 is a homolog of ATP6AP1 in yeast cells, and it plays a role in maintaining the intact function of V-ATPase. In addition to its function in acidifying vesicles or lysosomes, V-ATPase may act as a component of a signaling pathway that regulates autophagy through a mechanism independent of Tor/mTOR [99]. The Ccr4-Not complex stimulates the target of rapamycin complex 1 (TORC1) signaling, which activates ribosomal protein expression and inhibits autophagy by maintaining V-ATPase activity [100].

In addition to these mechanisms, cells have the capability to modify V-ATPase activity through the regulation of counterion conductance [101]. The presence of chloride channels serves to counteract the positive membrane potential that V-ATPases help to establish. A prime example of this can be seen with the H^+^/Cl^−^ antiporter ClC-5, which is localized in the apical vesicles of renal intercalated cells that are rich in V-ATPase activity. Disruptions in CLC5, as can happen with mutations, have been linked to Dent’s disease [63]. This condition is marked by a failure in endosomal acidification, leading to renal impairment.

In summary, the regulation of V-ATPase is critical for maintaining cellular homeostasis and responding to various physiological and pathological conditions. Understanding these regulatory mechanisms can help in the development of targeted therapies for CVDs associated with V-ATPase dysfunction.

## 5. Role of V-ATPase in the Cardiovascular System

V-ATPase plays a significant role in the cardiovascular system that extends beyond its well-known function in acidifying cellular compartments, contributing to various physiological and pathological processes. V-ATPase has been implicated in the regulation of inflammation, which is a critical factor in CVD. For example, it can influence the activity of immune cells like macrophages, which play a role in atherosclerotic plaque formation and the body’s response to vascular injury [26]. V-ATPase may also be involved in the regulation of vascular function, including the acidification of extracellular spaces in the vascular endothelium and smooth muscle cells, which could influence processes like angiogenesis and vascular remodeling [102]. In addition to its role in acidification, V-ATPase also has a protein sorting role in immature granules, which may also underscore its importance in cardiovascular health and disease [103]. Moreover, the fusion of autophagosomes with lysosomes does not depend on acidification by the V-ATPase [104,105].

### 5.1. Vesicle Loading and Coupled Transport

The regulation of V-ATPase activity directly influences vesicle loading and couples transport within cells, as the acidic environment created by V-ATPase is necessary for the proper functioning of many transport mechanisms. V-ATPase functions on the surface of lysosomes to regulate crucial cellular processes like receptor-mediated endocytosis and vesicular trafficking [106].

Upon mTORC1 activation, the peripheral V-ATPase V1 sector is sequestered in the cytoplasm, stabilized by its interaction with the chaperonin TRiC. Conversely, when mTORC1 activity is reduced, the V-ATPase V1 sector translocates to the lysosomal membrane, where it engages with the membrane-integral V-ATPase V0 sector to form the active proton pump. This assembly induces acidification of the lysosomal lumen, thereby enhancing proteolytic activity across the lysosomal population and triggering the degradation of accumulated proteins from both extracellular and intracellular sources [107]. V-ATPase is crucial for lysosomal acidification, a process that is essential for the transcriptional activation of the mitochondrial unfolded protein response (UPRmt). Under conditions of mitochondrial stress, the assembly of the V-ATPase complex on the lysosomal membrane leads to the activation of mTORC1 that results in the direct phosphorylation of the mitochondrial stress response (MSR) transcription factor, activating transcription factor 4 (ATF4), thereby initiating a cascade of events aimed at maintaining cellular homeostasis [108]. V-ATPase is necessary for amino acids to activate mTORC1 and guide it to the surface of the endosome/lysosome [109]. mTORC1 regulates the expression of V-ATPase and coordinates the regulation of TFEB phosphorylation and nuclear localization in a manner that is dependent on TFEB and V-ATPases [50]. Interestingly, EGF induces V-ATPase assembly by recruiting the extrinsic V1 subunits, and V-ATPase-regulated acidification mediates EGF-induced mTORC1 activation [38]. TMEM55B interacts with V-ATPase on the lysosomal membrane. In TMEM55B knockout cells, amino acid-induced phosphorylation of mTORC1 substrates S6K and 4E-BP is diminished, and the ability of the V1 subcomplex of V-ATPase to be recruited to the lipid rafts is inhibited. The absence of TMEM55B is also found to cause lysosomal stress, such as the translocation of the transcription factor TFEB to the nucleus. Taken together, TMEM55B contributes to the assembly of the V-ATPase complex in the lysosomal membrane lipid rafts and the subsequent activation of mTORC1 [41]. In addition, ATP6V1A has recently been implicated in coupling V-ATPases and autophagy activity via the mTORC1 complex [110,111].

### 5.2. pH Acidification and Sensing

The regulation of V-ATPase activity is intricately linked to pH homeostasis and sensing, which are essential for maintaining the cellular environment. V-ATPase is a critical enzyme that maintains cellular pH homeostasis and is involved in pH sensing, making it a key player in cellular physiology and disease. Understanding the complex interplay between V-ATPase, pH control, and cellular signaling is crucial for developing therapeutic strategies for diseases where V-ATPase function is disrupted. Its regulation and function are complex, involving intricate mechanisms that are still being unraveled by current research.

In the context of pH sensing, V-ATPase has been implicated as a potential sensor that regulates trafficking in the endocytic pathway. Its interaction with ARNO, the GDP/GTP exchange factor for ARF1 and ARF6, suggests that V-ATPase may be the long-sought pH-sensor in this pathway [73]. The V-ATPase a2 subunit (ATP6V0a2) is a crucial endosomal/lysosomal pH-sensor of V-ATPase [112]. In BI-1-overexpressing cells, V-ATPase was activated and lysosomal pH was lower than in control cells, and even when exposed to endoplasmic reticulum stress, BI-1 can maintain high levels of V-ATPase activity [113]. In RAW264 cells treated with chlorpromazine, V-ATPase maintains the luminal pH and protease activity in lysosomes/late endosomes, leading to the accumulation of phospholipids [114]. The V-ATPase-Ragulator complex acts as a primary cellular sensor in response to energy stress and doubles as a nuclear docking site by forming a V-ATPase-Ragulator-Axin/LKB1-AMPK complex, thereby orchestrating the metabolic switch between catabolism and anabolism [115].

### 5.3. Nutrient Signaling

Nutrient signaling is a critical cellular communication mechanism that allows cells to sense and respond to the availability of nutrients in their environment. This signaling pathway is essential for regulating cellular metabolism, growth, and differentiation. Nutrient availability can modulate the activity of various proteins, including the V-ATPase, which in turn can affect the cellular response to nutrient changes. For instance, under nutrient-rich conditions, the activity of V-ATPase may increase to facilitate the uptake and processing of nutrients, while under nutrient-poor conditions, its activity might be downregulated to conserve energy.

V-ATPase plays a crucial role in the amino acid-induced activation of mTORC1, a key regulator of cell growth that responds to the availability of nutrients and growth factors [109]. The interaction between V-ATPase and the Ragulator complex is amino acid-dependent, with the Ragulator complex facilitating the recruitment of mTORC1 to the lysosomal membrane during amino acid sensing [109,116]. In contrast, neutralization within the lysosome inhibits the amino acid-dependent changes that would otherwise lead to the assembly and reactivation of mTORC1 following periods of amino acid starvation [36]. Importantly, the loss of lysosomal acidification due to the inhibition of V-ATPase leads to an increase in the luminal concentration of most metabolites but has no effect on the concentration of most essential amino acids. In contrast, the inhibition of mTOR significantly reduces the lysosomal efflux of most essential amino acids, turning the lysosome into a cellular amino acid reservoir [117]. The lysosomal adapter protein Lamtor1, in conjunction with the lysosomal V-ATPase, constitutes an amino acid-sensing complex that acts as a platform for the activation of mTORC1 by amino acids; the interaction is essential for the M2 polarization of macrophages [118]. Importantly, ZNRF2 interacts with V-ATPase and positively regulates its function, thereby maintaining lysosomal acidity. As part of an amino acid sensing mechanism, ZNRF2 acts upstream of Rag-GTPases and V-ATPase to activate mTORC1 [62]. In addition, AKT is necessary for controlling the rapid response of lysosomal V-ATPase activity to changes in amino acid availability, and this response is dependent on the AKT isoform AKT2, which increases V-ATPase-dependent lysosomal acidification [119]. Recent studies in myocardium have indicated that the endoplasmic reticulum proton pump V-ATPase is a key enzyme in regulating the subcellular recycling of CD36, serving as a site for the interaction between various substrates and determining the cell’s substrate preference [10].

### 5.4. Others

In the context of LC3-associated phagocytosis, the assembly of the V-ATPase is a critical step that is mediated by reactive oxygen species (ROS), and this V-ATPase assembly is essential for the subsequent step of LC3 conjugation to the phagosome, highlighting a crucial role for ROS in the orchestration of this cellular process [120]. In addition, Golgi-associated LC3 lipidation requires V-ATPase in noncanonical autophagy [121]. The V-ATPase-ATG16L1 axis recruits LRRK2 through the WD40 domain of ATG16L1 and specifically mediates ATG8 lipidation on single membranes, playing a novel non-autophagic role in maintaining lysosomal homeostasis [122]. V-ATPase recruits ATG16L1 in a process known as V-ATPase-ATG16L1-induced LC3 lipidation [123].

V-ATPase may promote not only the degradation of Notch protein in lysosomes but also the activation of Notch signaling in endosomes through the acidification of early endosomes [124]. Thus, in signal-receiving cells, V-ATPase activity is required for Notch signaling. Interfering with V-ATPase does not prevent the lysosomal degradation of HIF1α, but rather leads to cellular iron consumption, thereby impairing prolyl hydroxylase (PHD) activity and resulting in HIFα activation. Iron supplementation directly restores the catalytic activity of PHD after V-ATPase inhibition, revealing an important link between V-ATPase, iron metabolism, and HIFs [125]. Recent research has found that quercetin, a small-molecule drug, can reverse iron deposition by regulating the Rab7-ATP6V1G1 axis [126].

In summary, V-ATPase plays a multifaceted role in the cardiovascular system, contributing to both physiological and pathophysiological processes. V-ATPase is a versatile enzyme with a range of functions in the cardiovascular system, from maintaining ionic balance and acid-base homeostasis to participating in cellular signaling pathways that regulate metabolism and growth. Its activity is critical for the normal function of the cardiovascular system, and its dysregulation can contribute to disease processes.

## 6. Dysfunction of V-ATPase-Dependent Lysosomal Acidification in Cardiovascular Diseases

**Figure 4:**
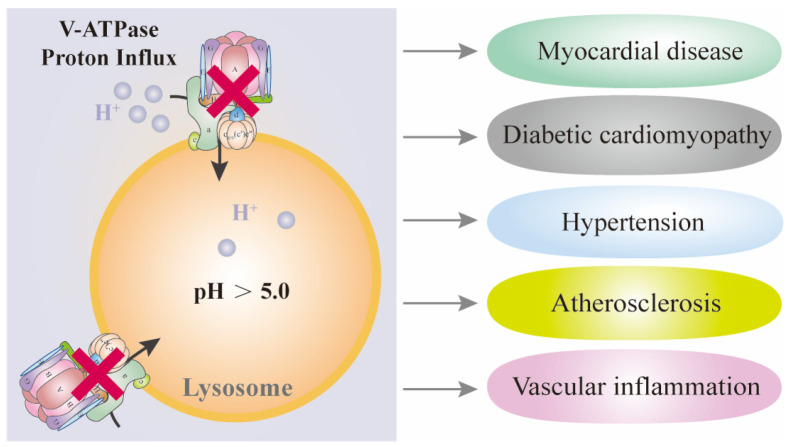
The primary cardiovascular consequences of dysfunctional V-ATPase-dependent lysosomal acidification. The dysfunctional lysosomal acidification can lead to myocardial disease, diabetic cardiomyopathy, hypertension, atherosclerosis, and vascular inflammation.

Dysfunction of V-ATPase-dependent lysosomal acidification can have significant implications for various cellular processes, including those in CVD. In the context of CVD, lysosomal dysfunction due to V-ATPase impairment could potentially affect several important processes. For example, lysosomes are involved in the inflammatory response, and their dysfunction could lead to chronic inflammation, which is a risk factor for various CVDs. Thus, CVD may benefit from V-ATPase-directed therapies. Figure 4 shows the primary cardiovascular consequences of dysfunctional V-ATPase-dependent lysosomal acidification.

### 6.1. V-ATPase-Dependent Lysosomal Acidification and Myocardial Disease

V-ATPase-dependent lysosomal acidification is a critical process that maintains the proper functioning of lysosomes, which are cellular organelles responsible for breaking down and recycling various biomolecules and organelles. In the context of myocardial disease, which is a disorder of the heart muscle often in response to various stressors, V-ATPase may play a significant role.

In cardiomyocytes, the depletion of RagA and RagB (RagA/B) culminates in a marked decrease in lysosomal ATP6V1B2 and ATP6V1D protein levels. This reduction triggers a decline in V-ATPase activity and disrupts lysosomal acidification, precipitating lysosomal dysfunction. Such dysfunction is ultimately implicated in the pathogenesis of hypertrophic cardiomyopathy and the manifestation of phenotypic lysosomal storage diseases [127]. Importantly, in RagA and RagB knockout cells, glutamine promotes the translocation of mTORC1 to lysosomes in a V-ATPase-dependent manner, and this process does not require Ragulator [128]. LAPTM4b-mediated mTORC1 activation requires active V-ATPase, indicating that LAPTM4b acts upstream of the H^+^ pump and RagA [129]. Cardiomyocyte-specific ablation of ATP6AP2 inhibits V-ATPase assembly, resulting in loss of V-ATPase function, which ultimately induces lethal heart failure [130].

The tissue deposition of amiodarone, an anti-arrhythmic drug, is the main source of side effects. Research has found that V-ATPase mediates the vacuolar sequestration of amiodarone, which will evolve into persistent autophagy and phospholipidosis. Although this type of cellular pathology is not cell-type specific, tissue macrophages seem to be particularly susceptible to it [131]. In myocardial cells treated with epirubicin, enforced expression of ATP6V0A2 restored lysosomal acidification and protected myocardial cells and mouse hearts from epirubicin-induced cardiotoxicity driven by ferroptosis [132]. The restoration of lysosomal acidification by ATP6V0A2 suggests a possible therapeutic strategy for countering the adverse effects of certain chemotherapeutic agents on cardiac function.

### 6.2. V-ATPase-Dependent Lysosomal Acidification and Diabetic Cardiomyopathy

V-ATPase-dependent lysosomal acidification plays a critical role in various cellular processes, including those implicated in diabetic cardiomyopathy. In the context of diabetes, there is an altered lipid metabolism that can lead to lipid accumulation in the heart. V-ATPase plays a role in regulating the acidification of endosomes and lysosomes, which are involved in lipid processing. Dysfunction in V-ATPase can impair lipid handling, contributing to cardiomyopathy.

A characteristic of diabetic cardiomyopathy is extensive lipid accumulation, which often leads to cardiac contractile dysfunction. The underlying mechanism involves a key role for v-ATPase. Specifically, excessive lipid supply to the heart leads to the disassembly of V-ATPase and the deacidification of endosomes/lysosomes [133]. Importantly, lysine/leucine/arginine supplements reduce cardiac lipid uptake by promoting the assembly of V-ATPase, increasing the possibility of treatment in conditions of lipid overload and related insulin resistance [134]. Additionally, recent research in the myocardium indicates that the endosomal proton pump V-ATPase is a site of interaction between various substrates, determining the cell’s substrate preference [10]. V-ATPase is crucial for the acidification of endosomes/lysosomes, which store lipid transporter proteins like CD36. An increase in lipid influx into cardiomyocytes can be sensed by V-ATPase, leading to its disassembly and causing endosomes/lysosomes de-acidification. This can result in the expulsion of CD36 to the cell surface, enhancing lipid uptake and contributing to lipotoxicity in the heart [135].

The modulation of V-ATPase activity through specific interventions, such as the supplementation with certain amino acids, has shown promise in reducing cardiac lipid uptake by promoting the assembly of V-ATPase. This suggests that V-ATPase activity modulation could be a potential therapeutic strategy to ameliorate lipid-induced cardiac dysfunction and fibrosis, indicating that V-ATPase may be a viable therapeutic target for diabetic cardiomyopathy.

### 6.3. V-ATPase-Dependent Lysosomal Acidification and Hypertension

In the context of hypertension, V-ATPase may play a role by influencing the delivery of lysosomal contents and their acidification, which in turn enables the breakdown of aggregates by lysosomal acid hydrolases and the cellular uptake of free cholesterol. This suggests that V-ATPase could be involved in the regulation of vascular function and the development of hypertension, potentially through its effects on lysosomal activity and cellular cholesterol homeostasis.

The (pro)renin receptor [(P)RR], a crucial component of the renin-angiotensin system, exerts a substantial influence on the progression of end-organ damage associated with hypertension [136,137]. A portion of the (P)RR, designated as ATP6AP2, functions as an auxiliary subunit of the V-ATPase in mammals. Compounds that block (P)RR have demonstrated positive effects in diabetic and hypertensive animal models by suppressing the tissue renin-angiotensin system [138].

While the exact mechanisms by which V-ATPase influences hypertension are not fully understood, its importance in maintaining lysosomal acidification and its implicated role in various human diseases highlight the need for further research into its potential as a therapeutic target for hypertension and related vascular disorders.

### 6.4. V-ATPase-Dependent Lysosomal Acidification and Atherosclerosis

Atherosclerosis is a disease characterized by the accumulation of lipids, cholesterol, and other substances in the arterial wall, leading to plaque formation and arterial narrowing. In the context of atherosclerosis, V-ATPase-dependent lysosomal acidification plays a crucial role in lipid processing and the prevention of lipid accumulation within cells. Dysregulation of V-ATPase activity has been linked to impaired lysosomal function, which can lead to the accumulation of lipids observed in atherosclerotic lesions. Furthermore, the acidification of lysosomes by V-ATPase also influences the cellular uptake of free cholesterol and the maintenance of cholesterol homeostasis, which is vital for preventing the development of atherosclerosis. Understanding the molecular mechanisms by which V-ATPase influences lysosomal acidification and contributes to atherosclerotic processes is therefore of significant importance for the development of novel treatments aimed at reducing the burden of this prevalent vascular disease.

Atherosclerosis has an inflammatory component. Lysosomes contain enzymes that can trigger inflammation if not properly regulated. V-ATPase dysfunction might lead to an uncontrolled release of these enzymes, contributing to inflammation and plaque formation. Macrophage organelle homeostasis is a critical factor in the development of cardiovascular diseases, such as atherosclerosis. Within this context, the absence of the lysosomal adapter protein Lamtor1, deprivation of amino acids, or the inhibition of V-ATPase and mTOR results in reduced M2 polarization and a corresponding increase in M1 polarization [118]. Additionally, the V-ATPase subunit ATP6V0d2 is essential for lysosomal acidification and is a key component of the macrophage-specific autophagosome-lysosome fusion mechanism, maintaining the homeostasis of cellular organelles in macrophages [139]. In blood macrophages, the lysosomal EAAT2 mediates the extrusion of lysosomal glutamate and aspartate to maintain the activation of the V-ATPase, thereby sustaining macropinocytosis and mTORC1 [140].

Dysfunction in V-ATPase could impair lipid processing, potentially leading to the accumulation of lipids observed in atherosclerosis. Phosphatidylinositol phosphate (PIP) directly interacts with isoforms of the V-ATPase subunits, determining the localization of V-ATPase subpopulations and participating in their regulation. The level of the vacuolar/lysosome-enriched signaling lipid PI(3,5)P2 affects the assembly and activity of V-ATPases containing the Vph1 a subunit isoform, thereby influencing lysosomal acidity [141]. Interestingly, PIP lipid binding could stabilize and activate V-ATPases in distinct organelles [142]. Lipid abnormalities observed in the plasma and liver in two recently discovered congenital metabolic defects caused by mutations in two V-ATPase assembly factors (TMEM199 and CCDC115) [143].

V-ATPase plays a significant role in lysosome-regulated calcium homeostasis. Dysregulation of calcium levels can affect vascular smooth muscle cell function and is implicated in atherosclerosis. PDK4 inhibits the interaction between V-ATPase and lactate dehydrogenase B, leading to reduced lysosomal degradation. It also blocks the nuclear translocation of TFEB, thereby suppressing lysosomal function. These changes lead to the disruption of autophagic flux, accelerate calcium deposition within vascular smooth muscle cells, and thus promote atherosclerosis [144].

It is important to note that while V-ATPase-dependent lysosomal acidification is integral to cellular function and could theoretically impact atherosclerosis, direct evidence linking the two processes is still emerging. Further research is necessary to fully understand the mechanisms by which V-ATPase might influence the development and progression of atherosclerosis.

### 6.5. V-ATPase-Dependent Lysosomal Acidification and Vascular Inflammation

Vascular inflammation is a key contributor to the pathogenesis of numerous cardiovascular diseases, including atherosclerosis, hypertension, and heart failure. Inflammatory cells, such as macrophages and endothelial cells, rely on lysosomal function to process and respond to inflammatory stimuli. When V-ATPase activity is compromised, lysosomal dysfunction can occur, leading to the uncontrolled release of inflammatory enzymes and cytokines. This dysregulation not only exacerbates local inflammation but also contributes to systemic inflammatory responses, further promoting vascular damage.

The IFITM3/V-ATPase-tetraspanin-VCP/p97 complex on the endolysosomes serves as a protein quality control and inflammation-inhibitory mechanism, which may be beneficial for therapeutic intervention in vascular inflammation [102]. The suppression of V-ATPase activity induces a significant G1 phase cell cycle arrest in human umbilical vein endothelial cells (HUVECs), triggers a G2/M phase arrest in human mammary epithelial cells (HMEC-1), and impedes endothelial cell migration, offering an attractive anti-angiogenic approach [145]. In HUVECs following HPS6 knockdown, as well as in endothelial cells from HPS6-deficient mice, Weibel–Palade bodies (WPBs) exhibit morphological abnormalities, a decreased number of WPBs is observed, and the tubulization of the von Willebrand factor (vWF) is compromised. Moreover, HPS6 interacts with ATP6V0D1, facilitating the transport of ATP6V0D1 to the WPB membrane where it assembles the V-ATPase complex to maintain the acidic pH of the WPB lumen, which is essential for the formation of vWF tubules during WPB maturation. Collectively, these findings suggest that HPS6 may play a role in the biogenesis of WPBs by being involved in the translocation of the V-ATPase to the WPB membrane [146].

In conclusion, the dysfunction of V-ATPase-dependent lysosomal acidification plays a significant role in the pathogenesis of cardiovascular diseases. Impaired lysosomal acidification disrupts essential cellular processes, including lipid metabolism, cholesterol homeostasis, and calcium regulation, all of which are critical for maintaining vascular health. The resulting dysregulation can lead to increased inflammation, cellular stress, and the progression of conditions such as atherosclerosis and myocardial disease. Given the pivotal role of V-ATPase in these processes, targeting its activity presents a promising therapeutic strategy for mitigating the adverse effects of cardiovascular diseases. Future research should focus on elucidating the precise mechanisms by which V-ATPase dysfunction contributes to cardiovascular pathology and exploring potential interventions that can restore lysosomal function and improve cardiovascular outcomes.

## 7. Conclusions and Perspectives

V-ATPase is a critical proton pump that maintains the acidic environment within lysosomes, which is essential for the proper functioning of these organelles in cellular degradation and recycling processes [147]. In the context of cardiovascular disease, V-ATPase-dependent lysosomal acidification plays a significant role. Dysfunction of V-ATPase can lead to impaired lysosomal function, which in turn can disrupt the balance between protein synthesis and degradation, leading to the accumulation of potentially toxic protein aggregates. Such disruptions have been implicated in various cardiovascular pathologies, especially atherosclerosis and heart failure. Identifying biomarkers of V-ATPase dysfunction could aid in the early diagnosis of cardiovascular disease or in monitoring treatment responses. Further research is needed to elucidate the precise molecular mechanisms by which V-ATPase contributes to cardiovascular disease. This includes understanding how V-ATPase dysfunction interacts with other cellular processes and how these interactions might be targeted therapeutically. Importantly, identifying specific components of the V-ATPase complex or related regulatory proteins that could serve as therapeutic targets is crucial. For example, small molecules that can modulate V-ATPase activity or restore its function in disease contexts are an area of active investigation. In addition, investigating how dietary and lifestyle factors might impact V-ATPase function and lysosomal acidification could potentially lead to interventions that could help prevent or mitigate cardiovascular disease.

Although the search results provided do not directly address the role of V-ATPase in cardiovascular diseases, we can infer some potential connections based on the known functions of V-ATPase and the broader implications of lysosomal dysfunction. It is important to note that while the connection between V-ATPase-dependent lysosomal acidification and cardiovascular diseases is not extensively covered in the search results, the potential impact of lysosomal dysfunction on cellular processes that are critical in the heart and vasculature could be significant. Further research is needed to fully understand the role of V-ATPase in cardiovascular health and disease.

Modulating V-ATPase-mediated lysosomal acidification presents several promising therapeutic avenues in CVDs. By targeting specific subunits of the V-ATPase complex, researchers are exploring ways to enhance lysosomal function in cardiomyocytes, potentially improving autophagy and reducing pathological protein accumulation. Small-molecule inhibitors and gene therapy approaches aim to restore pH balance within lysosomes, enhancing degradation of disease-related substrates. Additionally, researchers are investigating how modulating V-ATPase activity might influence inflammatory pathways and cellular signaling in cardiovascular contexts. While these approaches show potential, challenges remain in achieving tissue-specific targeting and understanding the complex regulatory mechanisms of V-ATPase in different cardiovascular pathologies.

In conclusion, this paper reviews the emerging roles of V-ATPase-dependent lysosomal acidification in CVD, offering insights that could inform future research and therapeutic development in cardiology. While our understanding of V-ATPase in cardiovascular disease is growing, there is still much to learn. Continued research in this area has the potential to not only advance our knowledge of disease mechanisms but also to identify new strategies for treatment and prevention.

## Figures and Tables

**Table 1:** Regulation of V-ATPase.

Type	Regulator	V-ATPase Activity	Location/Cell	Regulation Mechanism	Ref.
Regulated Assembly	Aldolase	-	Endosome/lysosome, HeLa cells	Aldolase engages with the transmembrane a subunit within the V0 sector and the soluble E and B subunits of the V1 sector	[29]
	Aldolase	↑	Yeast	Disruption of the binding between aldolase and the B subunit of V-ATPase results in disassembly and malfunction of V-ATPase	[30]
	The Ras/cAMP/PKA pathway	↑	Yeast	Active Ras2 blocks V-ATPase dissociation, PKA regulates V-ATPase assembly, and Active Ras2 affects the aldolase/V-ATPase interaction	[31]
	NDST3	↓	Human retina pigmented epithelial (RPE1) cells	Loss of NDST3 enhances the assembly of the V-ATPase holoenzyme on the lysosomal membrane via microtubule acetylation	[32]
	Glucose	↑	Yeast	Depriving the yeast cells of glucose, even for as little as 5 min, caused dissociation of approximately 70% of the assembled enzyme complexes into separate V1 and V0 subcomplexes	[33,34]
	Glucose	↓	Mammalian cells	Glucose starvation can enhance the assembly of V-ATPase via the AMPK and PI3K/Akt signaling pathways	[35]
	Amino acid	↑	HEK293T cells	Starvation for amino acids can enhance the assembly of the V-ATPase complex	[36]
	RAVE complex	↑	Yeast	The RAVE complex associates reversibly with V1 subcomplexes	[37]
	EGF	↑	Primary rat hepatocytes	EGF induces V-ATPase assembly	[38]
	RILP	↑	Hela cells	RILP recruits the ATP6V1G1 subunit to the membranes of late endosomes and lysosomes, and ensures the ATP6V1G1 stability	[39]
	PSEN1	↓	Neurons	PSEN1 plays a crucial role in facilitating the N-glycosylation process of the V0a1 subunit within the endoplasmic reticulum	[40]
	TMEM55B	↑	Neuro2A cells	TMEM55B interacts with V-ATPase to result in the assembly of the V-ATPase complex in the lysosomal membrane lipid rafts	[41]
	IGF2R	↓	C57BL/6 mice, bone marrow–nucleated cells, and THP1 cells	IGF2R induces Dnmt3a-mediated DNA methylation by activating GSK3α/β and subsequently blocks expression and assembly of V-ATPase	[42]
	HDAC6	↑	C57BL/6 mice, HT22	HDAC6 mediates V-ATPase assembly	[43]
Regulated Trafficking	AMPK	↓	The apical membrane in renal and epididymal cells; the renal cells	AMPK was found to block PKA-induced apical accumulation of the V-ATPase; AMPK directly phosphorylated subunit A	[44,45]
	cAMP	↑	The apical membrane in renal	The elevation of cAMP prompts the insertion of V-ATPase into the apical membrane, a process in which PKA-dependent phosphorylation of the subunit a plays a pivotal role	[46,47,48]
	PAT2	↑	Brown adipocytes	PAT2 facilitates the assembly of the lysosomal V-ATPase complex by bringing cytosolic V1 subunits to the lysosomal membrane	[49]
Regulation of Subunit Expression Levels	mTORC1	↑	Endosome/lysosome, mouse embryo fibroblasts	V-ATPases are regulated transcriptionally by mTORC1 through Tfeb	[50]
	Clathrin coat	↓	Mouse brain	Clathrin coat controls synaptic vesicle acidification by blocking vacuolar ATPase activity	[51]
	FBXO9	↓	A549 and H1299 cells	FBXO9 promotes the ubiquitination of subunit ATP6V1A to hinder the V-ATPase assembly	[52]
	Lamtor5	↑	Macrophages and peripheral blood mononuclear cells (PBMCs) from gender and age-matched systemic lupus erythematosus (SLE) patients	Lamtor5 is physically associated with ATP6V1A to promoting the V0/V1 assembly	[53]
	DDRGK1	↑	Mouse embryonic cells	DDRGK1 inhibits ubiquitin–proteasome-mediated degradation of V-ATPase subunits (including ATP6V0d1 and ATP6V1A) and maintains the stable expression of them	[54]
	miR-1	↓	Muscle tissue	miR-1 directly down-regulates the subunit vha-13/ATP6V1A via its 3′UTR	[55]
	RORα	↑	C57BL/6 mice	RORα induces the transcription of ATP6V1G1	[56]
	Prosapogenin A	↑	8505C and KHM-5M cells	Prosapogenin A significantly upregulates ATP6V1A, ATP6V1B2, and ATP6V0C	[57]
Post-Translational Modifications	The disulfide bond	↓	Bovine	The disulfide bond formed between cysteine 254 and cysteine 532 in the A subunit of bovine V-ATPase	[58]
Adjustments in Coupling Efficiency	Specific mutations in distinct non-homologous areas of subunit A	↓	Yeast	Specific mutations in distinct non-homologous areas of subunit A can enhance this coupling efficiency	[59]
	Subunit a isoform	↑	Yeast	-	[60]
Protein–Protein Interaction	ITM2A	↓	HEK293T cells	ITM2A interacts with V-ATPase to negatively regulate the activity of V-ATPase	[61]
	ZNRF2	↑	Mouse embryonic fibroblasts	ZNRF2 interacts with V-ATPase and positively regulates its function	[62]
Changes in Counterion Conductance	The ClC-5 chloride channel	↓	Active kidney cells	-	[63]

## Data Availability

Data are not available for this paper.

## Abbreviations

ATF4: Activating transcription factor 4; CVD, Cardiovascular disease; EGF, Epidermal growth factor; HMEC-1, Human mammary epithelial cells; HUVECs, Human umbilical vein endothelial cells; IGF2, Insulin-like growth factor 2; IGF2R, IGF2 receptor; LAMPs, Lysosomal associated membrane proteins; MSR, mitochondrial stress response; PHD, Prolyl hydroxylase; PIP, Phosphatidylinositol phosphate; (P)RR, (pro)renin receptor; RAVE, Regulator of the H^+^-ATPase of Vacuoles and Endosomes; RILP, Rab-interacting lysosomal protein; ROS, Reactive oxygen species; sAC, Soluble adenylyl cyclase; TORC1, Rapamycin complex 1; V-ATPase, Vacuolar-type ATPase; WPBs, Weibel-Palade bodies; vWF, von Willebrand factor; 3′UTR, 3′ untranslated region.
